# Analysing the implementation and transferability of an enhanced care pathway integrating systematic supportive cancer care and patient-partner support for people living with anal cancer: Protocol for a mixed-methods study in two French cancer centres

**DOI:** 10.1371/journal.pone.0359705

**Published:** 2026-10-23

**Authors:** Séverine Carillon, Rosie Faure, Maguy Del Rio, Claire Lemanski, Marguerite Tyran, Sandrine de Montgolfier, for the SOS – PP Group

**Affiliations:** 1 SESSTIM (Economic and Social Sciences of Health and Medical Information Processing), INSERM, IRD, Aix-Marseille University, Marseille, France; 2 Montpellier Cancer Institute (ICM), Montpellier, France; 3 Institut Paoli-Calmettes (IPC), Marseille, France; 4 Paris Est Creteil University, France; PLOS: Public Library of Science, UNITED KINGDOM OF GREAT BRITAIN AND NORTHERN IRELAND

## Abstract

**Background:**

Supportive cancer care (SCC) is essential for comprehensive oncology care. It addresses treatment-related adverse effects, reduces long-term sequelae and improves patients’ quality of life. Patient-partner (PP) programmes positively impact psychological well-being, patient empowerment and supportive care. Nevertheless, access to SCC remains inconsistent, fragmented and unequally distributed, while PPs are rarely integrated into routine cancer care pathways. Anal cancer is a predominantly localised cancer with high survival rates. Treatment is associated with acute toxicity and often severe long-term sequelae. This interventional public health research study aims to implement and evaluate an innovative enhanced care pathway combining systematic SCC and structured PP support for all individuals diagnosed with anal cancer living in France. The study will assess the intervention’s clinical, organisational, and social effects, and will identify the conditions required for its long-term sustainability and transferability.

**Methods and design:**

This is a longitudinal mixed-methods case study conducted in two French Comprehensive Cancer Centres. The intervention entails introducing a systematic, coordinated, and repeated SCC offer together with a structured PP support programme for all patients diagnosed with anal cancer and over 12 months following diagnosis. One centre will implement the enhanced care pathway, while the other will initially provide standard care before progressively adopting the intervention during the transferability phase. Quantitative data will include patient-reported outcomes, clinical information and implementation indicators. Qualitative data exploring implementation processes, organisational changes and contextual determinants, will be collected through observations, semi-structured interviews, focus groups and PP activity logs.

**Discussion:**

The study’s results will provide essential scientific knowledge on the implementation of programmes providing equitable SCC and PP support access to people living with anal cancer. Specifically, by combining implementation research, participatory approaches and mixed-methods evaluation, the project aims to identify the organisational conditions for the sustainable implementation of such programmes for this population, and their successful transferability to other populations living with cancer and to other healthcare settings.

## 1. Introduction

Recent advances in oncology have led to substantial improvements in cancer survival, both in terms of cure rates and longer life expectancy with the disease. However, cancer and related treatments have long-term consequences on the health-related quality of life (HRQoL) of patients and survivors. This has led to supportive cancer care (SCC) becoming an integral part of the care pathway. SCC is commonly defined as care and support provided for the prevention and management of the adverse effects of cancer and its treatment throughout the course of the disease. SCC is provided in conjunction with cancer specific treatments [1,2]. Its objectives are to reduce long-term sequelae and improve patients’ quality of life [3]. International guidelines recommend that the need for SCC be first assessed, that eligible patients be referred to the appropriate service(s) as early as possible (ideally at the time of diagnosis), and that they be reassessed throughout treatment and follow-up to ensure timely referral to appropriate services. A comprehensive, integrated and continuous approach to SCC is now widely recommended throughout Europe [1,4].

An ever-growing body of evidence demonstrates that SCC improves the management of cancer-related symptoms and treatment-related adverse effects, enhances HRQoL and, in some settings, increases overall survival [5–8]. SCC may also contribute to reducing long-term sequelae and may lower the risk of second primary cancers [9]. Studies have highlighted the benefits of integrated multidisciplinary SCC models addressing the physical, psychological and social dimensions of cancer care [10]. Likewise, interdisciplinary SCC and palliative care programmes delivered by specialised teams have been associated with improved symptom control, reduced healthcare utilisation and lower healthcare costs [11,12].

Despite these benefits, the practical organization and delivery of SCC remain complex [13]. Access to this care is often neither systematic nor equitable, and available services are frequently fragmented across healthcare settings. In the French context, the study country of the present work, the national health insurance system currently covers nine categories of SCC including pain management, nutritional support, psychological care, social and occupational support, adapted physical activity, fertility preservation, management of sexual health disorders, lifestyle counselling, and psychological support for relatives and caregivers [14]. Nevertheless, previous studies have reported considerable variability in the availability, organisation and accessibility of these services [15], together with persistent socioeconomic and geographical inequalities in access to supportive cancer care [16–19].

Alongside the development of SCC, at international level, there has been a growing focus on the contribution of patient partners (PPs) in oncology services. PPs are people who have experienced cancer themselves. They have acquired specific experiential knowledge related to living with the disease, the use of care services, and interactions with healthcare professionals (HCPs). This experiential expertise enables them to provide emotional, informational, and educational support to other patients [20]. Previous studies have shown that peer support delivered by PPs can reduce emotional distress and strengthen other patients’ coping skills [21,22]. Furthermore, evidence suggests that PP programmes can enhance HRQoL by encouraging healthy behaviours and mitigating symptoms of anxiety and depression [23,24]. Nevertheless, despite this increasing recognition of their contribution, PPs remain only partially integrated into routine oncology practice. Current related research has focused on the challenges of integrating PPs within multidisciplinary clinical teams and on defining their optimal role in the care pathway [25,26].

To date, few studies have explored how SCC and PPs can be systematically implemented, coordinated and sustained within routine oncology services. In particular, little is known about the organisational conditions required to ensure equitable access to SCC and to integrate PPs into multidisciplinary teams. Moreover, data on how to facilitate the transfer of such organisational models to different healthcare settings are scarce.

These challenges are particularly relevant in the context of anal cancer (AC), a relatively rare malignancy associated with a considerable physical, psychological and social (taboo) burden. Worldwide incidence continues to increase, largely owing to the growing prevalence of human papillomavirus (HPV) infections, the principal etiological factor for AC [27]. In France, approximately 2,000 new cases are diagnosed each year, predominantly in women (78%). The median age of cases is declining, standing at 62 years. Moreover, the affected population is socioeconomically heterogeneous [28].

Worldwide, most patients with AC are diagnosed with localized or locally advanced disease, while only 5–10% have metastatic disease at diagnosis. Standard treatment consists of six weeks of concomitant chemoradiotherapy (RTCT) [29]. The addition of induction chemotherapy before RTCT is currently being evaluated in clinical trials. Local recurrence rates are estimated at less than 20% for early-stage disease and between 20% and 40% for locally advanced tumours. Large cohort studies have reported a three-year overall survival probability of approximately 87.2% [30].

Despite these favourable survival outcomes, treatment is associated with acute toxicity and often severe long-term sequelae. Patients frequently experience debilitating sexual dysfunction, substantial psychological distress, severe pain during treatment, persistent fatigue, and urinary and faecal continence disorders throughout the care pathway [31–34]. Approximately 20% of patients require a temporary or permanent colostomy before or after treatment. Women of reproductive age are also at risk of treatment-induced infertility resulting from pelvic irradiation and may develop premature radiation-induced menopause.

Because of its treatment-related burden and significant long-term consequences, AC provides an appropriate clinical setting in which to evaluate an innovative French-based enhanced cancer care pathway combining both systematic SCC and PP. This intervention builds upon international recommendations advocating systematic, repeated, coordinated, and patient-centred supportive care throughout the cancer continuum [4]. It also incorporates structured PP support with the aim of improving patients’ HRQoL and reducing the long-term negative impact of treatment.

The overall objective of the present study – named SOS-PP (*Soins Oncologiques de Support et Patients partenaires* in French or SCC & PP in English) – is to evaluate the implementation of this enhanced pathway for individuals diagnosed with AC, to assess its clinical, organisational, and social effects, and to identify the conditions required for its long-term sustainability and transferability.

The study has been co-designed since its inception by HCPs, researchers, and PPs. It is therefore grounded in the principles of participatory health research, in which experiential and scientific knowledge are jointly mobilised throughout the research process [35,36].

## 2. Materials and Methods

### Study objectives

The SOS–PP study is structured around four specific objectives:

(1) To analyse the implementation of an enhanced care pathway combining the systematic provision of SCC with structured PP support.
(2) To describe the organisational and social effects of this pathway, to evaluate its effects on participants’ HRQoL, and to assess the reduction of adverse event reductions.
(3) To identify the structural, organizational and financial conditions for the sustainability and transferability of this enhanced care pathway.
(4) To describe and analyse the implementation of participatory research process throughout the project.

### Study design

This interventional public health research study is designed to evaluate, at a care facility level, the implementation, effects and transferability conditions of an enhanced care pathway for patients living with AC [37].

It comprises a longitudinal mixed-methods case study, combining clinical, organisational, and social analyses. It will be conducted over two years in two French Comprehensive Cancer Centres (CCC): an intervention site, where the enhanced care pathway will be implemented, and a comparison site, where routine care will continue throughout the evaluation period (i.e., 2 years). During the final year of the project, the intervention will be adapted and transferred to the comparison site.

A mixed-methods design was selected as exclusively quantitative or qualitative approaches cannot fully capture the dynamics of the implementation of complex organisational interventions such as this one [38]. Qualitative methods will explore how the intervention operates in routine practice, the specific organisational and contextual conditions it requires to be successful, its organisational and social effects, and the factors and conditions influencing its transferability to other healthcare settings. Quantitative methods will provide a detailed description of the study population and the cancer care cascade while measuring the clinical and social effects of the enhanced care pathway compared with routine care.

### Study setting

The study will be conducted in two CCC which are private, non-profit institutions in France, exclusively dedicated to cancer care, research, and education.

#### Intervention site.

The intervention site is one of the largest referral centres for AC in France. SCC is already integrated early into routine outpatient oncology in this centre through a dedicated multidisciplinary supportive care unit, which brings together specialised supportive care professionals within a single organisational structure. Patient partnership has also become progressively embedded in routine clinical practice. An increasing number of trained PPs at this CCC contribute to patient care pathways, participate in structured support programmes, and receive financial compensation for these activities [39,40].

#### Comparison site.

At the comparison site, SCC is currently offered in routine clinical practice and primarily depends on needs identified by physicians during medical consultations.

This CCC has a longstanding commitment to patient-centred care and health democracy. Patient partnership is a nascent strategy in the structure. So-called volunteer “patient witnesses” occasionally provide peer support when requested by healthcare professionals, but no systematic PP programme has yet been implemented.

### The Enhanced Cancer Care Pathway

The intervention–an enhanced cancer care pathway—consists of introducing a systematic, coordinated, and repeated offer of SCC together with structured PP support throughout the first year following diagnosis. The pathway begins at the end of the diagnostic consultation. All patients undergo a systematic SCC needs assessment conducted by a radiation oncology nurse. The enhanced care pathway will extend throughout the first year of treatment and follow-up, covering the twelve months following the pre-treatment consultation.

#### Systematic SCC.

At the intervention site, every newly diagnosed patient with AC will be routinely offered SCC in the dedicated service at three predefined time points:

- T1: before cancer treatment initiation;
- T2: four months after treatment completion (approximately seven months after the pre-treatment consultation);
- T3: twelve months after the pre-treatment consultation.

#### *PP support*.

At the same three time points, patients will be systematically offered support from a trained PP. In addition, PPs will make a scheduled follow-up telephone call approximately two weeks after treatment, a period during which patients frequently experience severe treatment-related toxicities and psychological distress.

In summary, during the first year following diagnosis, patients will be able to access the multidisciplinary SCC unit on three occasions and receive PP support on four occasions (Fig 1).

**Fig 1:**
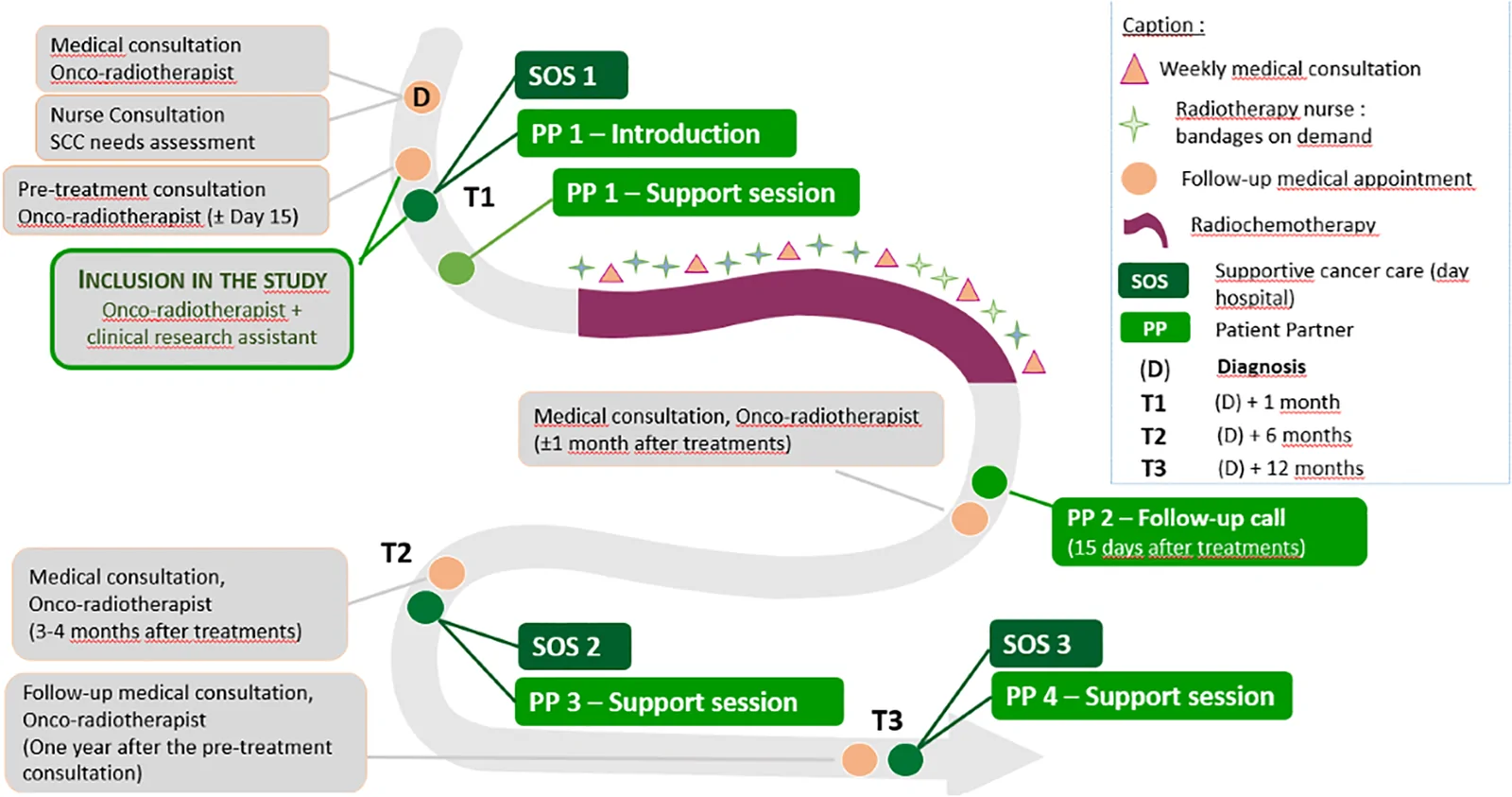
Intervention: Enhanced care pathway for patients with anal cancer.

Importantly, the management of the pathology itself will remain unchanged. The intervention exclusively concerns the organisation and delivery of SCC, provided by the usual multidisciplinary HCPs —including physicians, nurses, dietitians, psychologists, and social workers, etc. —as part of the intervention centre’s current routine clinical practice. Services will be delivered by the existing dedicated SCC unit (see above) where three or four coordinated consultations will be scheduled consecutively according to patients’ previously assessed needs. This organisational model promotes multidisciplinary coordination while minimising the burden of multiple appointments.

PP support will be provided by a PP specifically recruited and trained to liaise with the SCC unit. Following an initial face-to-face meeting between the patient and the PP, subsequent contact may take place either in person or remotely, according to patients’ preferences.

### Conceptual Framework

To examine equitable access to SCC and PP, we shall use the access to healthcare framework developed by Levesque and colleagues [41]. This framework considers access as the dynamic interaction between healthcare services and patients. It encompasses both healthcare system characteristics—including approachability, acceptability, availability and accommodation, affordability, and appropriateness—and patients’ ability to perceive healthcare needs, seek care, reach services, pay for care, and engage with HCPs (see Fig 2).

**Fig 2:**
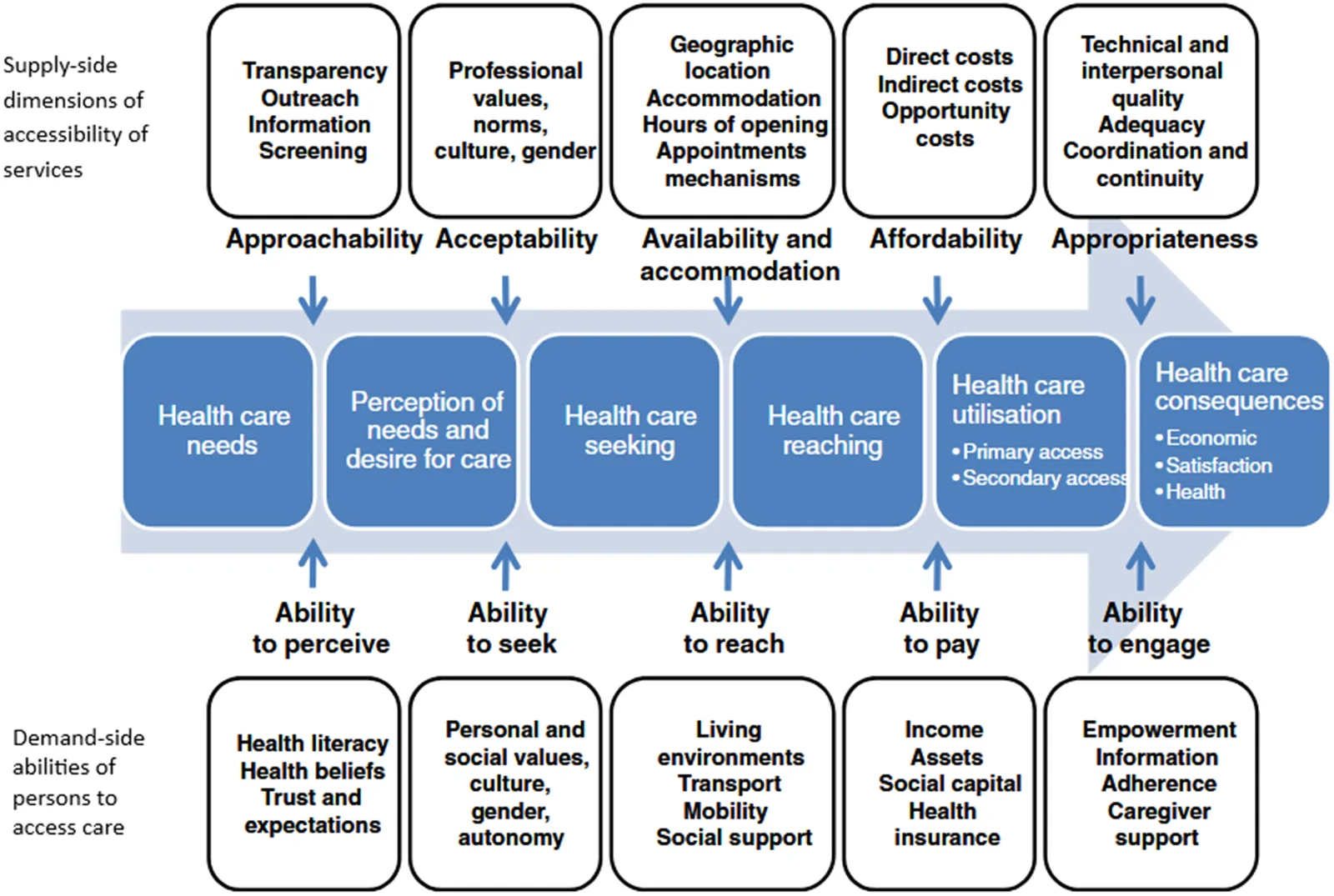
Conceptual Framework (Levesque et al., 2013).

By integrating both the supply and demand dimensions of healthcare, this conceptual framework provides a comprehensive model for understanding access to SCC and PP programmes. It also ensures that the structural, organisational, and individual factors that facilitate or hinder implementation can be examined.

Since its publication in 2013, the Levesque framework has been widely applied across diverse healthcare settings and populations to investigate healthcare access from both patient and provider perspectives [42]. In the present study, we hypothesise that the enhanced care pathway will improve equitable access to SCC and PP support by addressing barriers operating at multiple levels of the healthcare system.

### Study hypotheses

Drawing on Levesque’s conceptual framework [41] together with current evidence regarding SCC and PP interventions [8,43–45], the study is based on three main hypotheses (see Fig 3).

**Fig 3:**
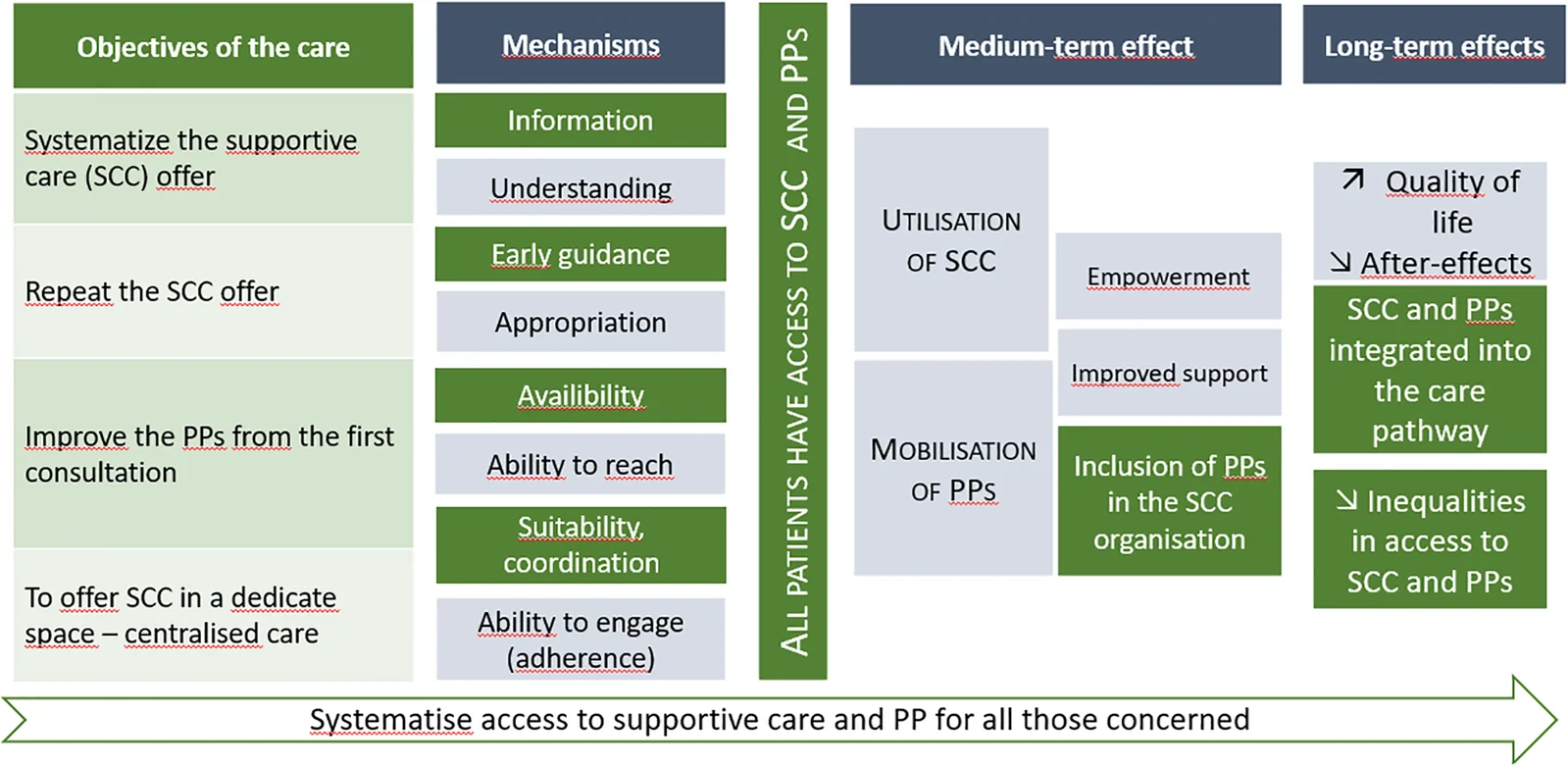
Intervention theory.

1. Providing a systematic, repeated, and coordinated offer of SCC combined with structured PP support will increase access to these two types of services for all individuals diagnosed with AC, thereby reducing social inequalities in access to supportive care.
2. The enhanced pathway will facilitate timely provision of information, earlier referral to appropriate supportive care services, better understanding and uptake of available supportive care, greater patient engagement with supportive services, and finally, improved patient empowerment and self-management capacities throughout the cancer trajectory.
3. Over the longer term, systematic integration of SCC and PPs will strengthen their incorporation within cancer care pathways and contribute to improved HRQoL and reduce treatment-related sequelae.

### Participants

#### Eligibility criteria.

Eligible participants are adults aged 18 years or older diagnosed with histologically confirmed squamous cell carcinoma of the anal canal, initiating treatment at one of the two participating CCCs, and able to provide informed consent. Exclusion criteria are inability to communicate in French, and cognitive impairment preventing informed participation in the study procedures.

#### Sample Size.

Approximately 50 and 30 patients living with AC are managed annually at the intervention and comparison sites, respectively. Based on previous clinical experience, we anticipate that approximately 80% of all eligible patients will agree to participate in the study and complete the first supportive care assessment (T1).

Accordingly, recruitment targets are 40 participants per year at the intervention site and 20 participants at the comparison site. The total study population will therefore comprise 120 participants enrolled over the two-year recruitment period.

This relatively high expected participation rate reflects several factors: the substantial SCC needs associated with AC, the existing established multidisciplinary Supportive Care Unit at the intervention site, carefully designed patient information procedures, and the limited burden the study will impose on participants.

#### *Recruitment procedures*.

Potential participants will be identified during routine oncology consultations. The study will be introduced verbally by the treating radiation oncologist during either the first oncology consultation or the pre-treatment consultation. Patients expressing an interest in participating will receive a paper-based information sheet explaining the study objectives, procedures, data protection measures, and participant rights. Patients may provide verbal informed consent either during the same consultation or at a subsequent visit.

Following consent, participants will meet a Clinical Research Associate (CRA) who will confirm their eligibility, assign a unique study identification number, answer any remaining questions regarding study participation, and complete the inclusion procedure. Participants will then be formally enrolled immediately after the pre-treatment consultation and followed prospectively for 12 months. The recruitment procedures will be fully integrated into routine clinical care in order to minimise participant burden and maximise inclusion while guaranteeing routine medical practice.

Patient recruitment will take place over a two-year inclusion period, followed by one year of follow-up for all participants.

### Data collection

A convergent mixed-methods design will be used to analyse the implementation of the enhanced care pathway, its effects, and the conditions required for its sustainability and transferability (See Table 1).

**Table 1:** Summary.

Objectives	Sub-objectives	Data to be collected	Methods	investigative techniques	dimensions/ analytical framework
**1.** To analyse the implementation of an enhanced care pathway combining the systematic provision of SCC with structured PP support	• Analyse the context • Analyse processes • Highlight the experience of those involved • Highlight the structural, organisational and relational conditions at the local and institutional levels for this integration	Structural and organisational context Components and dynamics of the intervention Subjective experience of HCPs, PP and patients	Qualitative	Observations Interviews Logbooks	Consolidated Framework for Implementation Research (CFIR)
	• Describe and analyse the participating population • Describe the use of the proposed combined SCC and PP support pathway	Socio-demographic profiles Clinical data Medical follow-up data PP support follow-up data	Quantitative	Patient questionnaire Clinical and follow-up questionnaire Logbook	Effective use of SCC and PP by participants
2. To describe the organisational and social effects of this pathway, to evaluate its effects on participants’ HRQoL, and to assess the reduction of adverse event reductions.	• Describe and analyse social and organizational effects the enhanced care pathway	Patient’s living environment, disease experience and care pathway	Qualitative		Levesques et al.
	• Describe and measure the clinical effects of the enhanced care pathway	Changes in patients’ quality of life, psychological distress, sequalae, and their ability to cope with cancer	Quantitative	Questionnaires (EQ5D5; QLQ-ANL27; HADS; Cancer box)	Quality of life Decreased sequelae
**3.** To identify the structural, organizational and financial conditions for the sustainability and transferability of this enhanced care pathway	• Identify the structural and organizational conditions for the sustainability of the improved care pathway • Identify transferability challenges, specifically, identify elements and mechanisms of this pathway to be replicated or adapted • economic evaluation: comparison of the costs of this innovative strategy with the routine strategy	Stakeholders’ perceptions of the organisational effects of the intervention; facilitators and barriers to its implementation; lessons learned throughout implementation and anticipated challenges; components of the intervention Direct medical costs use of supportive care and PP’s support	Qualitative Quantitative	Observations Focus groups clinical and follow-up questionnaires and information supplied by the accounting services of the care centres	Consolidated Framework for Implementation Research (CFIR)
4. To describe and analyse the implementation of participatory research process throughout the project.	• Identify the conditions for involving PPs and HCPs in participatory research. • Describe and analyse the effective implementation of participatory research.	stakeholder engagement, collaborative decision-making, integration of PPs’ experiential knowledge into research activities, evolution of stakeholder partnerships over time.	Qualitative	Meeting minutes and observational notes analysis Observations Interviews	*To be defined*

#### Quantitative data.

*Questionnaires:* The quantitative data will contribute to the achievement of two study objectives; (1) the analysis of the pathway’s implementation, and (2) the analysis of its effects. Two types of questionnaires will be used: a patient questionnaire and a clinical and follow-up questionnaire.

*Patient questionnaire:* To characterise the study population, participants will self-administer a patient questionnaire using a tablet computer. A paper version will be available if requested. This questionnaire will collect sociodemographic information and data describing their living environment, including sex, age, postal code of residence, marital status, educational attainment, employment status, and other relevant social determinants of health.

To evaluate the effects of the enhanced care pathway on patients’ health status and HRQoL, four validated instruments will be administered at each assessment time point:

- EQ-5D-5L, to measure HRQoL;
- EORTC QLQ-ANL27, to assess the effects of the pathway on disease/treatment sequelae and their evolution;
- Hospital Anxiety and Depression Scale (HADS), to evaluate psychological distress;
- CASE-Cancer (Communication and Attitudinal Self-Efficacy Scale for Cancer), to assess how well patients are coping with AC.

Together, these instruments will provide a comprehensive assessment of patients’ physical, psychological, and functional outcomes throughout the first year following diagnosis.

*Clinical and follow-up questionnaire:* Clinical data will be collected by a CRA from participants’ electronic medical records. Variables will include date of diagnosis, dates of all (i.e., scheduled and unscheduled) consultations, treatment, support care provided, date of inclusion in the study and dates of scheduled follow-up visits, etc. These data will help create a detailed description of participants’ clinical profiles and trajectories, the evolution of their health status, and documentation of the implementation of the enhanced care pathway.

*Timing of Quantitative Assessments:* Both questionnaires will be administered at three predefined time points corresponding to key stages of the care pathway:

- T1: within one month after the pre-treatment consultation and before treatment initiation;
- T2: four months after treatment completion (approximately seven months after the pre-treatment consultation);
- T3: twelve months after the pre-treatment consultation.

#### Qualitative data.

Qualitative methods will address four complementary objectives: understanding the intervention’s implementation processes (Objective 1); exploring its organisational and individual effects (Objective 2); identifying conditions for its sustainability and transferability (Objective 3); documenting the study’s participatory research process (Objective 4). Data collection will combine observations, interviews, focus groups, and structured field diaries and logbook.

*Intervention implementation: Process Evaluation and Effects (Objectives 1&2):* The intervention’s implementation will be explored through an in-depth field study using a combined inductive and empirical approach designed to capture everyday clinical practices and stakeholders’ experiences.

The different stages of the pathway will be observed by the researcher responsible for the qualitative study using a field notebook. This researcher will collect, among other things, data on the context, data on factors that facilitate and hinder the intervention’s implementation, interactions between HCP teams, the PP and patients, and data on the perceived influence of these dynamics on the care experience.

In addition, in-depth and repeated semi-structured interviews, aimed at gathering stakeholders’ experiences) and views on the care pathway (improved vs. routine) will be conducted with the HCPs involved and with participating patients (n = 20).

The PP will keep an ad-hoc logbook to identify both the number of planned and actual follow-up support sessions with each participant, and the content of support provided, for example the topics discussed, difficulties encountered, etc. The logbook’s contents were developed by adapting the logbook used for the Parole-Onco project in Quebec [23].

The data collected on the intervention’s implementation will provide insights into the conditions needed to ensure its transferability to other cancer care settings.

*Sustainability and Transferability Evaluation:* The organisational conditions required for sustaining and transferring the intervention will be investigated through observations of pathway implementation meetings, together with two rounds of focus groups conducted approximately one year apart at each study site. Participants will include HCPs, hospital administrators and PPs involved in the project (PP involved in the care pathway and PPs involved in the committee).

The focus groups will address stakeholders’ perceptions of the organisational effects of the intervention, facilitators and barriers to its implementation, acceptance of the enhanced care pathway, lessons learned throughout implementation and anticipated challenges related to transferability, and components of the intervention to keep or adapt for its future implementation.

Focus-group discussion guides will be specifically developed for this study using a participatory co-design approach.

*Evaluation of the Participatory Research Process:* Throughout the project, researchers will conduct participant observations during team meetings. Meeting minutes and observational notes will be analysed using a reflexive approach to document stakeholder engagement, collaborative decision-making, integration of PPs’ experiential knowledge into research activities, and evolution of stakeholder partnerships over time.

This analysis will aid understanding of how participatory governance influences the implementation and transferability of complex health interventions in general.

*Qualitative study implementation modalities:* Patients participating in qualitative interviews will be purposively selected to ensure a diversity of socio-demographic profiles (age, gender, socio-professional category, etc.). The total number of interviews conducted will be determined by data saturation, defined as the point at which no new theme emerges from successive interviews (44).

Whenever possible, interviews will be conducted immediately after clinical appointments at T2 - four months after treatment completion – and T3 - twelve months after the pre-treatment consultation (Fig 4). Otherwise, appointments will be scheduled according to participants’ preferences, either in person in the CCC or remotely by telephone.

**Fig 4:**
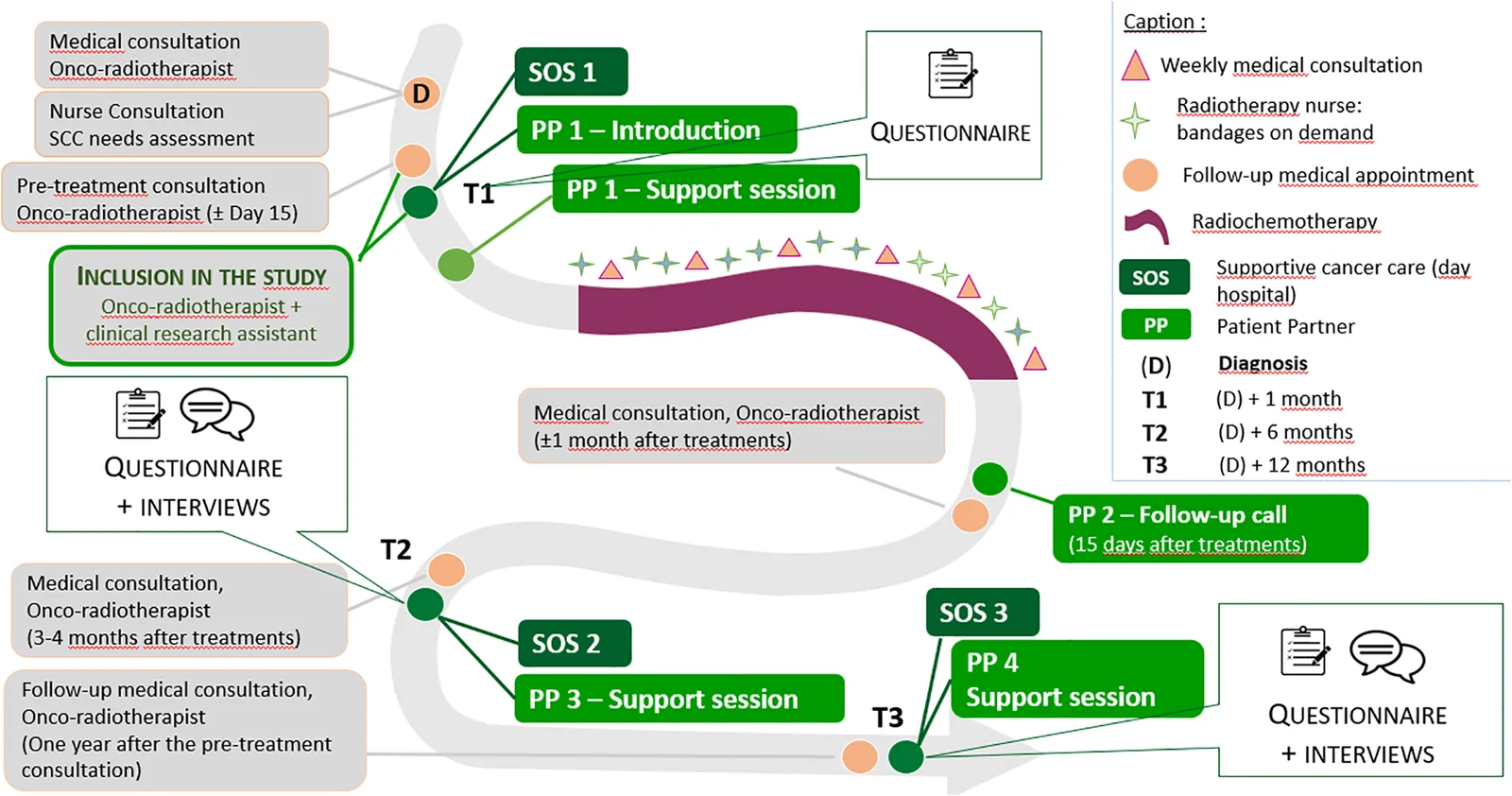
Data collection and intervention phase.

HCPs will be interviewed at their workplace or at another location of their choice.

All interviews will be conducted by an experienced qualitative researcher using interview guides specifically developed and co-designed for this study. With participants’ permission, interviews will be audio-recorded using encrypted, non-networked digital recorders. Recordings will be transcribed verbatim, pseudonymised, and permanently deleted after transcription.

The focus groups will be conducted on site. With participants’ consent, discussions will be audio- and video-recorded, and complementary field notes will be taken.

PP will complete her activity diaries through a secure online platform. All qualitative data will be pseudonymised before analysis.

### Data analysis

#### Qualitative analyses.

Qualitative analyses will be based on the complete transcription of semi-structured interviews, focus groups, observations and the PP’s activity logs. All transcripts will be pseudonymised before coding and analysis.

The analysis will combine inductive thematic analysis with a theory-informed implementation analysis. Initial coding will allow themes to emerge directly from participants’ narratives. These inductively-generated themes will subsequently be interpreted using the Consolidated Framework for Implementation Research (CFIR) to provide a systematic understanding of the intervention’s implementation processes [46]. Particular attention will be paid to organisational context, contextual facilitators and barriers, the role of social actors [47], adaptations introduced during implementation (whether planned or unplanned) [48], interactions between HCPs, PPs and patients, and finally, mechanisms influencing the pathway’s sustainability and transferability. The analysis of intervention- induced effects will focus on stakeholders’ reports of the direct or indirect effects of the intervention on the lives of the persons concerned and on the organisation of related care services (48).

Two complementary analytical approaches will be undertaken. First, a cross-case thematic analysis will identify recurring themes across interviews and focus groups, enabling comparison of experiences between stakeholder groups (HCPs, patients, etc.) and study sites. Second, a case-oriented analysis will examine each participant’s experience, which in turn will provide us will a global perspective. This will enable us to interpret individual care trajectories within their broader organisational and social context.

Participant observations will be analysed alongside interview data to triangulate observed practices with participant-reported experiences. This triangulation will strengthen the credibility of the findings and provide a more comprehensive understanding of the intervention’s implementation.

*Analysis of Transferability:* Transferability will be examined throughout the implementation process (36) rather than being assessed solely at the end of the study. The transferability analysis will draw upon evidence generated during the first phases of implementation, including utilisation of SCC services, uptake of PP support, effects identified on HRQoL and disease sequelae, organisational facilitators and barriers, and contextual characteristics of both participating CCCs.

Particular attention will be given to identifying the components of the intervention that are essential for its successful implementation, those which will require adaptation for different healthcare settings, and the organisational conditions needed to ensure the pathway’s sustainable integration into routine oncology practice.

The principal indicator of successful transferability will be the effective implementation of the initial SCC consultation (T1), with a target participation rate of 80% among eligible patients at the transfer site. The secondary criterion will be the improvement in HRQoL and the reduction of sequalae twelve months after treatment initiation.

#### *Quantitative Analyses*.

*Statistical analyses:* Statistical analyses will cover descriptive statistics (average and standard deviations, medians, averages and standard deviations). The comparability of the two CCC samples’ characteristics (age, gender, stage of cancer at diagnosis, etc.) will be tested using a gap-reduced test or a Chi² test if the application conditions are met. We shall also conduct stratified analyses by institution, sex and gender, using a Chi2 test and t-tests if the conditions for their application are met. If necessary, we will use non-parametric statistical tests.

*Integration of Mixed-Methods Findings:* Quantitative and qualitative findings will first be analysed independently before being integrated through methodological triangulation. Integration will seek to identify convergence between quantitative outcomes and qualitative experiences, complementary explanations for observed implementation outcomes, discrepancies requiring further interpretation, and contextual mechanisms explaining implementation success or failure.

This integrated analytical strategy is expected to provide a comprehensive understanding of how, for whom, and under which organisational conditions the pathway can be successfully implemented, sustained and transferred to other oncology settings.

*Medical-economic analysis:* In order to estimate the cost of the enhanced care pathway and the economic conditions for its transferability (Objective 3), an economic evaluation will be carried out, as well as a comparison of the costs of this innovative strategy with the routine strategy (i.e., SCC only) used in current practice.

To take into account patients’ HRQoL, the efficacy criterion used in the medico-economic evaluation will be QALY (quality-adjusted life year), obtained by multiplying the life years by a quality-of-life score (utility score). For this purpose, the utility score, which reflects the patient’s preference for the state of health in question, will be estimated in the cost-utility analyses using the standard generic scale EQ-5D-5L, in accordance with the recommendations of France’s High Authority of Health. (49) This utility score and QALYs will be estimated at different points in the data collection (T1, T2 and T3). In addition, the HADS questionnaire assessing anxiety and the QLQ-ANL27 questionnaire assessing the psychological consequences and distress related to living with AC, which both be administered at the T1, T2 and T3.

The direct medical costs borne by the hospital (nurses, consultations, day hospital for support care: physiotherapists, dieticians, etc.), will be integrated into the analysis. Specifically, these costs will be identified on the basis of both clinical and follow-up questionnaires administered by the ARC and information supplied by the accounting services of the care centres. The sources of unit prices will be derived from hospital databases. The average total cost of each strategy (i.e., the enhanced pathway versus routine care) will be estimated by multiplying the quantities consumed for each type of care listed above by the unit price of each type of care.

An assessment of the total cost of the innovative strategy, including support care, conducted at the interventional site will be carried out on the basis of these data, as well as an inventory of the practice carried out at the comparison site, in order to be able to study the transferability of this strategy to the comparison site taking into account the constraints inherent in this site.

### Data management plan

The secure Voxco platform for creating and managing online surveys and databases will be used to capture data from questionnaires, and to organize and centralize data. All data will be stored on this platform for the duration of the data collection. At the end of the data collection (i.e., two years of recruitment followed by 12 months of follow-up for each participant), the pseudonymised data will be transferred and stored on the INSERM network’s secure server until the end of the study and two years later for publications. All these data will then be destroyed by the provider (Voxco). A data warehouse will then be used to store the data once the study has been completed and the final results have been published.

### Patient and public involvement and engagement and governance

The SOS-PP project was conceived and co-designed by clinicians, researchers and PPs. Several governance bodies systematically involving researchers, HCPs and PPs will be set up for the project. A comprehensive coordination committee will bring together the leaders of each stakeholder group (i.e., PPs, HCPs, researchers) to ensure comprehensive monitoring of the study. A local steering committee will bring together different stakeholder types involved in the project at each of the two participating sites to monitor operational progress. These two committees will meet together once a year to share experiences at each site and collectively discuss issues of sustainability and transferability of the improved care pathway. Finally, a committee of PPs already involved in the monitoring committee but not participating in the SOS-PP study, will be responsible for objectively monitoring the project and participating in the interpretation of the results of each study phase, and in the process of transferability of the intervention. Bringing their lived experience of cancer and their experiential knowledge to each stage of the project, the members of this committee have been remunerated for their participation since its inception.

### Ethical considerations

This protocol has been submitted to the Ethics Committee. It received approval from the Committee for the Protection of Persons (CPP- Ile de France XI) on 16 January 2025 (ref: 2024-A01644-43). All research procedures were conducted in accordance with the principles outlined in the Declaration of Helsinki.

Prior to participation, eligible persons will receive both oral and written information about the study. They will be invited to read a hand-delivered paper-based information leaflet. All information on the conduct of the research, data protection and patients’ rights can be found in this leaflet. Patients will be included only if they did not express any objection to participation. This will be collected prior to inclusion by the HCP proposing participation.

This study was conducted as Category 3 research involving human participants (RIPH 3) under French law (Loi Jardé). For this type of study, verbal consent (instead of written) is required. Participants were provided with clear and appropriate information about the study objectives, the data collected, and their right to object to participation. Inclusion was based on the absence of objection. This information and non-opposition procedure was approved by the Ethics Committee (CPP- Ile de France XI).

At the intervention site, the SCC pathway is already part of routine care. Consequently, people who do not wish to participate in the SOS-PP study will experience no change in their current care pathway. At the comparison site, routine care (i.e., no systematic SCC and no PP support) will continue irrespective of study participation during the three years of data collection.

All data collected in this study will be pseudonymised: each patient included will be assigned an identifier number. This pseudonymization will be preserved during and after the study, as well as when the results are published.

### Study status and timeline

This project will take place over 4 years (see Table 2).

**Table 2:** Data collection timeline and intervention phase.

	Year 1 (2025)				Year 2 (2026)				Yea 3 (2027)				Year 4 (2028)			
PROJECT STEPS	Q1	Q2	Q3	Q4	Q1	Q2	Q3	Q4	Q1	Q2	Q3	Q4	Q1	Q2	Q3	Q4
**Intervention**			**Deployment of experimental on-site**											**Transferability**		
**Preparation**																
Administrative and regulatory procedures																
Training of PP and ARC, digitalisation of questionnaires																
**Data collection**																
Participant inclusion																
Clinical and follow-up questionnaires																
Patient questionnaires																
Intervention site observations																
Interviews with patients in sites																
Interviews with HCPs																
Focus groups comprising HCPs and PPs on both sites																
**Data analysis**																
Qualitative																
Quantitative																
Meeting to prepare for on-site transferability																
Transferability workshop with stakeholders of the 2 sites																
**Valuation**																
Publication, participation conferences																

Specifically, the study of the enhanced care pathway will span two years. The collection of quantitative and qualitative data will take place over the first 3 years (2 years of inclusion and 1 year of follow-up). Finally, the collection of medico-economic data will take place after one year and then after two years. The fourth year of the project will be devoted to disseminating the findings of the study and transferring the enhanced pathway to the comparison site.

At the time of protocol submission, thirty patients had been included in the intervention site and 7 in the comparison site. The first participant was enrolled on April 7, 2025. The estimated completion date for data collection is April 2028 (2 years of inclusion and 1 year of follow-up). Analyses are scheduled to begin in January 2028, with results expected by October 2028.

## 3. Discussion

The SOS-PP study will provide unique insights into the conditions for the implementation and transferability of the proposed innovative enhanced care pathway, which comprises a comprehensive approach to care that combines systematic SCC and PP support, with a view to greater care equity for persons living with a CA. It will develop knowledge on the conditions for implementing this pathway, and the conditions for its transferability to other cancer healthcare contexts. This study will also complement existing literature on peer support. It will provide a detailed and contextual knowledge of the organisational, structural and temporal dimensions of this novel comprehensive, intersectoral and interdisciplinary healthcare offer. It will also contribute, using a health promotion approach, to general research on the transferability conditions of a public health intervention, by tackling the question of how to adapt and transfer an intervention initially developed and evaluated in a singular context to one or more culturally and structurally heterogeneous different contexts, while maintaining its effectiveness.

### An intervention that takes into account social inequalities in health

This intervention study shall seek to maximise the chances of reducing disease sequalae and long-term treatment adverse effects, and of improving the HRQoL of patients, taking into account persons’ living conditions and socio-demographic characteristics. The consideration of social inequalities in health is core to the intervention and to the SOS-PP study, as reflected in several elements: (1) the intervention has been organised in accordance with the principle of proportionate universalism: while the offer of systematic SCC and PP support is intended to reach all patients diagnosed with AC, the specifics of the intervention may vary from one individual to another depending on their needs; (2) the study is based on a research which addresses all persons concerned by the health problem in question, with data collection incorporating the social determinants of health; (3) the study design ensures that knowledge produced from more than one research site, with heterogeneous populations and contexts, will be used; (4) the study and intervention include intersectoral collaborations.

### Study limitations

This study will involve a relatively small number of patients (n = 120) which limits its scope. In addition, the enhanced care pathway includes the intervention of only one PP, which means that study results cannot be generalised for other healthcare settings. However, data triangulation will help to limit these limitations.

### Perspective: fostering an integrated approach to SCC and PP in other cancers and institutions

The results of the SOS-PP study will engender several benefits at the CCC level: (i) the development of an enhanced care pathway that is evaluated, coordinated, adapted and permanently integrated into the existing care pathway of patients living with AC, in which support by PP will be systematically offered at key time points; (ii) the adaptation of the enhanced care pathway and its implementation in another CCC; (iii) the development of operational recommendations to support the sustainability of the enhanced care pathway, and its transferability to other care services and healthcare settings, such as colorectal cancer.

Moreover, this project will provide valuable scientific knowledge on the integration of systematic SCC in current AC care pathways, and on the development of the patient partnership at the level of a care pathway. Depending on the results of this study, one possible avenue for exploration would be to develop an integrated approach to SCC and PP for other cancers and institutions.

## 4. Declarations
